# Viability, Stability and Biocontrol Activity *in Planta* of Specific *Ralstonia solanacearum* Bacteriophages after Their Conservation Prior to Commercialization and Use

**DOI:** 10.3390/v14020183

**Published:** 2022-01-19

**Authors:** Belén Álvarez, Laura Gadea-Pallás, Alejandro Rodríguez, Begonya Vicedo, Àngela Figàs-Segura, Elena G. Biosca

**Affiliations:** 1 Departamento de Microbiología y Ecología, Universitat de València (UV), 46100 Valencia, Spain; mariabelen.alvarez@madrid.org (B.Á.); lauragadeap@gmail.com (L.G.-P.); arodriguez@darwinbioprospecting.com (A.R.); bvicedo@camn.uji.es (B.V.); angela.figas@uv.es (À.F.-S.); 2 Departamento de Investigación Aplicada y Extensión Agraria, Instituto Madrileño de Investigación y Desarrollo Rural, Agrario y Alimentario (IMIDRA), 28800 Madrid, Spain; 3 Departamento de Ciencias Agrarias y del Medio Natural, Universitat Jaume I (UJI), 12071 Castellón, Spain

**Keywords:** plant pathogenic bacterium, phage, bacterial wilt, biological control, lyophilization

## Abstract

*Ralstonia solanacearum* is a pathogen that causes bacterial wilt producing severe damage in staple solanaceous crops. Traditional control has low efficacy and/or environmental impact. Recently, the bases of a new biotechnological method by lytic bacteriophages vRsoP-WF2, vRsoP-WM2 and vRsoP-WR2 with specific activity against *R. solanacearum* were established. However, some aspects remain unknown, such as the survival and maintenance of the lytic activity after submission to a preservation method as the lyophilization. To this end, viability and stability of lyophilized vRsoP-WF2, vRsoP-WM2 and vRsoP-WR2 and their capacity for bacterial wilt biocontrol have been determined against one pathogenic Spanish reference strain of *R. solanacearum* in susceptible tomato plants in different conditions and making use of various cryoprotectants. The assays carried out have shown satisfactory results with respect to the viability and stability of the bacteriophages after the lyophilization process, maintaining high titers throughout the experimental period, and with respect to the capacity of the bacteriophages for the biological control of bacterial wilt, controlling this disease in more than 50% of the plants. The results offer good prospects for the use of lyophilization as a conservation method for the lytic bacteriophages of *R. solanacearum* in view of their commercialization as biocontrol agents.

## 1. Introduction

*Ralstonia solanacearum* [1,2] is a phytopathogenic bacterium that mainly affects crops such as potato, tomato, eggplant and pepper, and some ornamental species such as geranium, producing vascular bacterial wilt disease and potato brown rot in tubers [3,4,5]. This pathogen has been reported as one of the most destructive in the world [4], due to its global extension and the importance of the damaged crops, which are basic for human nutrition. It is also considered a quarantine bacterium in many countries, and a select agent in the USA [6,7,8].

This bacterial species was a member of the former “*Ralstonia solanacearum* species complex” presently divided into the closely related *R. pseudosolanacearum*, *R. solanacearum* and *R. syzygii* subsp. *indonesiensis* [2].

The infection usually occurs through the roots, since the pathogen is soil and water borne. Once inside the plant, *R. solanacearum* multiplies in the cortex and xylem, ascending to the aerial parts [9,10,11]. External symptoms produced by *R. solanacearum* in the host are mainly wilting, growth retardation, adventitious roots in the stem, and epinasty in the leaves [12]. Normally the disease progresses until plant death, due to xylem obstruction and the destruction of surrounding tissues.

The great ability of *R. solanacearum* to survive in different environments that can constitute reservoirs for long periods, such as plant debris, water or soil, without altering the pathogenic character of the bacterium, makes it difficult to control of the disease in the field [13,14,15]. Physical and chemical methods developed to eradicate *R. solanacearum* from affected crops, as the use of fumigants and/or cupric compounds, showed variable results, besides having an impact in soils and contributing to the appearance of bacterial resistance [10,14]. Biological control such as the use of bacteriophage viruses can be an alternative to these methods, particularly by lytic bacteriophages (phages), since they can destroy the bacterial host. Their antibacterial potential against phytopathogenic bacteria allows their use as agricultural biocontrol agents in an environmentally respectful way [15,16,17,18].

Due to this potential, an efficient method of preservation and distribution is required, especially if the phages are to be marketed after large-scale production for field application. Lyophilization is one of the most commonly used conservation methods for microorganisms [19]. It allows them to be preserved for long periods while maintaining their viability. It is carried out in three phases, namely, the freezing of the sample to be treated, followed by drying that leads to the sublimation of the ice crystals [20,21]. Applied to phages, the cost of transport and maintenance is reduced [21,22] but, freeze drying is a great stress for viruses, since it can alter their survival and/or their lytic capacity, because it affects their structure, destabilizing it, especially protein compounds such as the capsid [23]. For this reason, lyophilization should be carried out with cryoprotective agents, which have the function of physically and/or chemically stabilizing the compounds to be lyophilized in the freezing and drying processes [24]. There are many types of cryoprotectants. For bacteriophages or different types of viruses, the most common are disaccharides such as saccharose or trehalose [20,22,24,25,26,27]. Lower loss of viability was observed with trehalose and saccharose as cryoprotectans compared to mannitol and polyethylene glycol immediately after lyophilization and in long-term storage [24]. The use of saccharose or polyethylene glycol favored bacteriophage stability following lyophilization only at high concentrations [25]. In other cases, soluble starch, maltose, glucose, skim milk, peptone, gelatin, glycerol, PBS, NaCl, and the combinations of some of them at different concentrations were also used, with losses of viability of around 99.9% with glucose, gelatin, PBS and NaCl [22,25]. With respect to *R. solanacearum* phages, no data on the conditions for a successful lyophilization have been reported so far.

Recently the foundations of a new biotechnological control method were established based on three *R. solanacearum* lytic phages called vRsoP-WF2, vRsoP-WM2 and vRsoP-WR2 with specific activity against the pathogen and biocontrol efficiency *in planta* [28,29,30,31]. However, with a view to their commercialization, there were some unknown aspects, such as the survival capacity and the maintenance of the lytic activity of these phages after subjecting them to a conservation method such as lyophilization. In this work, the viability and stability of the phages preserved by this method with various cryoprotectants against various strains of *R. solanacearum* were determined for different periods. In addition, the efficiency of the lyophilized phages in the biological control of the strain CFBP 4944 of this pathogen in susceptible tomato plants was also evaluated.

## 2. Materials and Methods

### 2.1. Bacterial Strains and Culture Conditions

The *R. solanacearum* strains used in this work are in Table 1. Among them, the strain CFBP 4944 was the only one used in all the tests. The strains were kept in cryopreserved stocks at −80 °C with 25% (*v*/*v*) glycerol and were grown in Casaminoacids Peptone Glucose (CPG) medium (casaminoacids 0.1%, peptone 1%, glucose 0.5%) [32], with bacteriological agar (1.5%). The general medium Luria–Bertani (LB) (1% NaCl, 0.5% yeast extract, 1% tryptone) [33] was used as the liquid culture medium. The semiselective medium from South Africa (SMSA) (0.1% casaminoacids, 1% peptone, 0.5% glycerol, 1.5% agar, crystal violet 0.0005%, bacitracin 1250 U/L, polymyxin sulfate B 600,000 U/L, penicillin 825 U/L, chloramphenicol 0.0005%, triphenyltetrazolium chloride 0.005%) [34] was used in some assays carried out with the strain CFBP 4944. SMSA is a semiselective medium for *R. solanacearum* isolation.

The culture conditions of the bacterial strains were the following: in liquid medium, overnight cultures (16–18 h) in 5 mL of LB and incubation at 28 °C with shaking (120 rpm), and in solid medium, incubation at 28 °C for 48–72 h.

### 2.2. Phages and Amplification Conditions

In the different assays, three specific *R. solanacearum* phages, vRsoP-WF2, vRsoP-WM2 and vRsoP-WR2, isolated and purified from environmental water from different regions of Spain [28,29,30,31] were used. The three phages were separately kept with their bacterial host *R. solanacearum* strain CFBP 4944 in cryopreserved stocks at −25 °C in 25% (*v*/*v*) glycerol.

For the amplification of the phages, overnight cultures of *R. solanacearum* strain CFBP 4944 were performed and the optical density (OD) at 600 nm was adjusted to a value of 0.5, using a spectrophotometer (Thermo Scientific Genesys 20, Waltham, MA, USA). Then aliquots from the bacterial cultures were separately inoculated with each of the three phages and were incubated overnight at 28 °C with shaking (120 rpm). After the incubation period, phage lysates were obtained for each of the phages, which were kept at 4 °C until subsequent assays.

Before using the phages, each phage lysate was centrifuged at 10,000 rpm for 10 min, the supernatant was filtered through a sterile 0.22 µm pore diameter filter, and the titration of the supernatant was performed by the double layer agar method. For this, serial ten-fold dilutions were made in SM buffer (50 mM TrisHCl pH 7.5, 100 mM NaCl, 10 mM MgSO_4_ and 0.01% gelatin) [35]. Volumes of 100 µL of the dilutions were mixed with volumes of 200 µL of a suspension of the strain CFBP 4944 (OD_600 nm_ 0.5), and these mixtures were inoculated into CPG top agar (or soft CPG) tubes that were poured onto solid CPG medium. The plates were incubated at 28 °C for 24–48 h, and then the plaques formed were counted to calculate the titer of the phage as plaque-forming units (PFU)/mL.

### 2.3. Stability Assays of One Single Phage Lyophilized with Various Cryoprotectants

Suspensions of phage vRsoP-WF2 intended for lyophilization were initially prepared with three cryoprotective agents: 50% glycerol [36], and 0.1 M and 0.5 M saccharose and trehalose [22,37].

The suspensions of vRsoP-WF2 in the different cryoprotectants were distributed in sterile glass vials for lyophilization (200 µL per vial) and frozen for 1 h at −80 °C while the lyophilizer was tempered at −51.5 °C. The vials with the frozen viral suspensions were introduced in a lyophilizer (Virtis/Benchtop, Virtis SP Scientific) operating at a vacuum pressure of 15.9 mTorr for 18 h [21]. Subsequently, the lyophiles were then sealed with a vacuum pressure of 1.7 mTorr, and stored at 4 °C until use.

The viability of phage vRsoP-WF2 after lyophilization with different cryoprotectants was calculated according to the following formula:

Viability = (Initial V/Final V) × 100

with “Initial V” being the number of PFU/mL before lyophilization, and “Final V” the number of PFU/mL after lyophilization and resuspension of the phages. Titration was performed in triplicate after rehydration of the viral particles in the same initial volume of 200 μL of sterile distilled water by the double layer agar method as abovementioned.

### 2.4. Stability Assays of One Single Phage Lyophilized or Conserved at 4 °C over Time

To compare the stability (that is, maintenance of viability over time) of the phage vRsoP-WF2 after lyophilization with respect to conservation at 4 °C, suspensions of the phage were simultaneously prepared either with saccharose and trehalose 0.5 M for lyophilization, or in SM buffer and the liquid culture medium LB for conservation at 4 °C. Stability was monitored during the storage period of vRsoP-WF2 lyophiles and the nonlyophilized suspensions after 0, 15, 30 and 60 days. Plaque counts were performed as indicated above.

### 2.5. Stability Assays of the Three Phages Lyophilized with One Cryoprotectant

To proceed with the lyophilization of phages vRsoP-WF2, vRsoP-WM2 and vRsoP-WR2, 0.5 M trehalose was selected as the cryoprotective agent. Thus, 1/100 dilutions of the phage stocks were performed in 0.5 M trehalose to obtain a concentration of 10^8^ PFU/mL, and 200 µL of each of the viral suspensions were transferred to sterile glass vials. Lyophilization procedure was as abovementioned. Then, viability and stability of the three phages at different times were determined. Phage lyophiles were resuspended in 200 µL of sterile distilled water and titrations were performed by the double layer agar method. Phage viability was determined by calculating the difference between the titers (PFU/mL) of each of the lyophilized phages at each time “Final V”, and those of the nonlyophilized phages used as controls “Initial V”.

At the same time that the viral suspensions were disposed for lyophilization, additional aliquots were prepared for the three phages in LB liquid medium and in SM buffer, which were kept at 4 °C to compare viability and stability under different conservation methods.

Titrations were performed for the three phages, either lyophilized or kept at 4 °C in LB medium and in SM buffer at 0, 1, 2, 5, 8 and 12 weeks of storage in these conditions. All the assays were performed in triplicate.

### 2.6. Effect of Lyophilization on the Lytic Activity of the Three Phages

To determine the influence of lyophilization on the activity of the *R. solanacearum* phages, qualitative spot assays of bacterial lysis were performed with a collection of phage-sensitive *R. solanacearum* strains listed in Table 1. Bacterial suspensions were prepared in phosphate-buffered saline (PBS) 10 mM pH 7.2 for each of the different strains, which were spread onto the surface of CPG plates. Then, drops of 5 µL of the viral suspensions of each of the nonlyophilized phages (controls) and each of the phages lyophilized after 1 and 4 weeks were inoculated onto the surface of the same plates. The observation of lysis in the spot areas was recorded after 24–48 h at 28 °C to detect lytic activity. The assays were performed in triplicate for the three phages.

### 2.7. Biocontrol Assays in Planta

#### 2.7.1. Susceptible Host Plants and Growing Conditions

For the biocontrol assays, tomato plants (*Solanum lycopersicum* L.) variety Roma, sensitive to *R. solanacearum* [13,38,39] were used.

Seeds were sown using vermiculite as a substrate, and cultivated for 30 days before the biocontrol assays. Since vermiculite requires the administration of nutrients for the correct plant development, they were watered with Hoagland’s nutrient solution [40] throughout the experimental period. The plants were kept during the assays in a plant growth chamber (Panasonic) at 26 °C, with 12 h light and 8 h darkness for 15 days, under conditions of biological containment.

#### 2.7.2. Biocontrol Assays with the Three Phages

Inoculations were performed with *R. solanacearum* and/or the mixture of the three phages either nonlyophilized or lyophilized.

The strain CFBP 4944 was grown in LB liquid medium for 16–18 h and the suspensions adjusted to OD_600 nm_ = 0.1 (approximately 10^8^ CFU/mL), then serial tenfold dilutions were made to a final concentration of 10^5^ CFU/mL for plant inoculation. Regarding phage suspensions, the concentration of each one was adjusted to 10^9^ PFU/mL, and for mixtures, phage suspensions were combined at a final concentration of 10^8^ PFU/mL. For the *in planta* inoculations, suspensions were prepared with mixtures of the bacterium and the three phages at a final bacterium:phage concentration of 10^5^ CFU/mL:10^8^ PFU/mL. Positive and negative controls were included, consisting of *R. solanacearum* suspensions at the same final concentration, and PBS or mixtures of the three phages either lyophilized or nonlyophilized, respectively. The assays were carried out in sets of 10 tomato plants per treatment, in triplicate.

For inoculations, a bevel wound was made on the stem of each plant with a sterile scalpel, between the cotyledons and the first petiole, and volumes of 5 µL of each mixture, suspension or PBS were applied. Inoculated plants were incubated in a growth chamber (Panasonic, M2R-352-PE) for 15 days, and the appearance of bacterial wilt symptoms was daily monitored.

Wilting symptoms in less than 25% of leaves were recorded as first symptoms to be confirmed in subsequent evaluations. Progressive increase in wilting incidence to 50–75% of the leaves and branches was recorded as positive wilting in the plant. More than 75% of leaves and branches affected were recorded as near collapse or collapse of the wilted plant [28].

From symptomatic plants, *R. solanacearum* reisolation was performed to confirm the presence of the pathogen. Thus, stem samples above the inoculation point were taken, superficially disinfected, and cut up in PBS buffer to obtain the plant extracts. Direct platings were made on SMSA and incubated at 28 °C for 48–72 h. Colonies with *R. solanacearum* morphology were purified and PCR-identified according to [41].

Plants were also analyzed for phage detection. The obtained plant extracts were filtered through 0.22 µm pore membranes and mixed with *R. solanacearum* suspensions adjusted to OD_600 nm_ = 0.5, then the mixtures were added to melted CPG top agar, which was poured onto CPG plates and incubated at 28 °C for 24–48 h.

### 2.8. Statistic Analysis

Data (PFU/mL) from the viability and stability assays of the phages either lyophilized or preserved in LB medium or in SM buffer at 4 °C, which were performed in triplicate, were processed by an analysis of variance ANOVA with the GraphPad Prism 6 program after their logarithmic transformation. For some assays, a multinomial analysis using a Bonferroni post hoc analysis was also used with SPSS 20.0 software (IBM SPSS Statistics, Chicago, IL, USA). *p* values ≤ 0.05 were considered statistically significant.

## 3. Results

### 3.1. Viability and Stability of Phage vRsoP-WF2

#### 3.1.1. Phage Viability after Lyophilization

**Figure 1:**
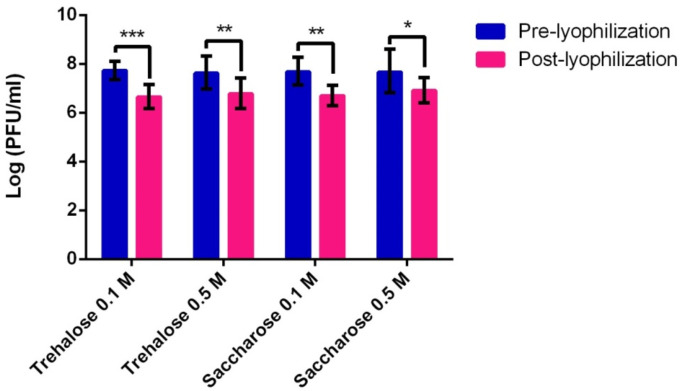
Effect on viability of phage vRsoP-WF2 of lyophilization with saccharose or trehalose at 0.1 M or 0.5 M. Titration was carried out by the double layer agar method on CPG plates. Data are the means for assays performed at least in triplicate, and error bars indicate variation as the standard deviation. Asterisks above bars denote statistically significant differences between the two signaled columns: * *p* < 0.05, ** *p* < 0.01, *** *p* < 0.001.

To investigate the effect of the lyophilization process on the survival and viability of the phage vRsoP-WF2, three cryoprotectants were initially tested: 50% glycerol, 0.1 M and 0.5 M saccharose, and 0.1 M and 0.5 M trehalose, the titre of the phage being determined before and after lyophilization. The results obtained in this initial test are shown in Figure 1. Phage vRsoP-WF2 did not survive the lyophilization process when 50% glycerol was used as a cryoprotective agent or, at least, survival fell below the detection limit (not shown). On the contrary, saccharose and trehalose made it possible to recover viable viral particles in high numbers at the two concentrations tested, these being higher in the case of 0.5 M for both disaccharides, with respect to the initial phage suspension. Thus, vRsoP-WF2 counts after lyophilization were from 3.50 × 10^7^ PFU/mL in the control to 3.01 × 10^6^ and 4.65 × 10^6^ PFU/mL in 0.1 M and 0.5 M trehalose, and to 3.26 × 10^6^ and 7.24 × 10^6^ PFU/mL in 0.1 M and 0.5 M saccharose, respectively. Differences were statistically significant (*p* ≤ 0.05) between initial and final viabilities for both cryoprotectants at both concentrations but, not when comparing the two cryoprotectants to each other, at both concentrations.

Based on these data, saccharose and trehalose at 0.5 M were selected to repeat the test. The results confirmed the favorable vRsoP-WF2 survival after lyophilization with these two cryoprotectants. Phage counts were from 2.35 × 10^7^ PFU/mL in the control to 5.90 × 10^6^ PFU/mL and 1.74 × 10^6^ PFU/mL in the lyophiles prepared in 0.5 M trehalose and 0.5 M saccharose, respectively. The reduction in viability of phage vRsoP-WF2 was less than a logarithmic order in trehalose, contrarily to saccharose. Between the two cryoprotectants, there were about three times more recovery of viable viral particles with trehalose with respect to saccharose. Although these differences in viability between the two cryoprotectants were not statistically significant, there were statistically significant differences (*p* ≤ 0.05) when comparing initial and final phage suspensions only in the case of saccharose, meaning that the loss in viability was not significant when trehalose was used as a cryoprotectant. Therefore, 0.5 M trehalose could be a better choice than 0.5 M saccharose.

On the other hand, the appearance of the lyophiles prepared in 0.5 M saccharose was less compact and more crystalline than in 0.5 M trehalose, which were more compact and with a gritty aspect.

#### 3.1.2. Phage Stability after Lyophilization

**Figure 2:**
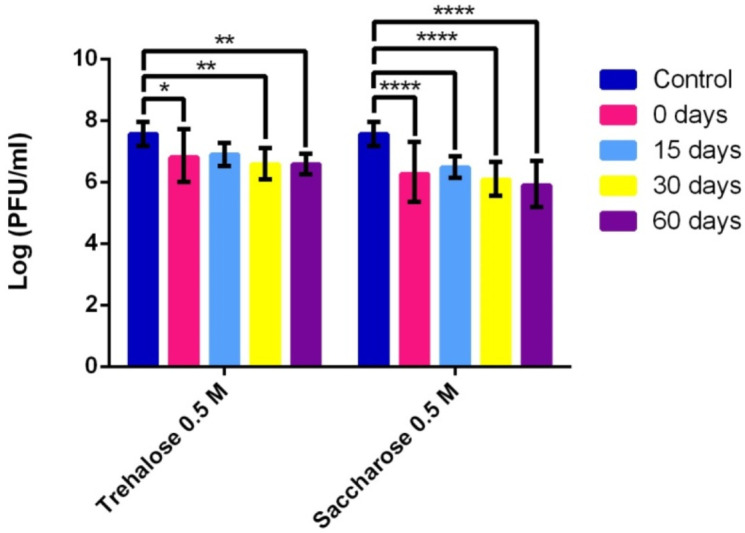
Stability of phage vRsoP-WF2 lyophilized with trehalose or saccharose at 0.5 M and stored at 4 °C over time. Titration was carried out by the double layer agar method on CPG plates. Data are the means for assays performed at least in triplicate, and error bars indicate variation as the standard deviation. Asterisks above bars denote statistically significant differences between the two signaled columns: * *p* < 0.05, ** *p* < 0.01, **** *p* < 0.0001.

The results for lyophiles of vRsoP-WF2 prepared in 0.5 M trehalose and in 0.5 M saccharose kept at 4 °C showed sustainment in the counts throughout the experimental period of 60 days (Figure 2), after an initial decrease of around one log unit, and a subsequent slight decrease. In most cases differences were statistically significant (*p* ≤ 0.05) between initial viability values and values obtained at each time with both cryoprotectants (Figure 2). Stability was greater in 0.5 M trehalose than in 0.5 M saccharose, with a final concentration of viable viral particles of about 10^6^ PFU/mL and 10^5^ PFU/mL respectively at the end of the experimental period. These differences between the two cryoprotectants were statistically significant (*p* ≤ 0.05). Therefore, trehalose as a cryoprotectant in lyophilization allowed not only a greater survival of the vRsoP-WF2 phage lyophiles but, also a greater stability of the phage particles, at least in the first 2 months after lyophilization.

**Figure 3:**
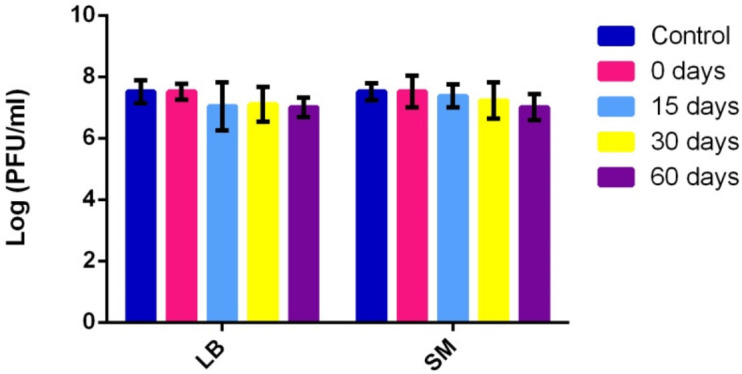
Stability of phage vRsoP-WF2 suspensions preserved in LB medium and in SM buffer at 4 °C over time. Titration was carried out by the double layer agar method on CPG plates. Data are the means for assays performed at least in triplicate, and error bars indicate variation as the standard deviation. No statistical differences were found.

When the stability of the lyophilized vRsoP-WF2 phage particles kept at 4 °C was compared with that of the phage suspensions of the controls kept in LB medium and in SM buffer at 4 °C, the results showed greater viability and stability of the viral particles in the nonlyophilized suspensions throughout the same experimental period (Figure 3).

The viability of the vRsoP-WF2 phage suspensions did not undergo initial decreases as observed when the viral particles were lyophilized. In fact, the titre of these suspensions did not drop below the initial logarithmic order (10^7^ PFU/mL) throughout the 60 days and, although there were small decreases in the concentration of viable viral particles over time, these differences were not statistically significant. Therefore, high stability of phage vRsoP-WF2 in LB medium and in SM buffer was observed. Comparing both media, counts of vRsoP-WF2 stabilized in LB medium after 15 days while in SM buffer a smooth but more progressive decrease was observed. Although values were slightly higher in SM, these differences were not statistically significant.

### 3.2. Viability and Stability of Phages vRsoP-WF2, vRsoP-WM2 and vRsoP-WR2

#### 3.2.1. Phage Viability after Lyophilization

To verify the viability and survival of the three phages over time after lyophilization, the viral suspensions were titrated before and after this process with 0.5 M trehalose as a cryoprotective agent.

**Figure 4:**
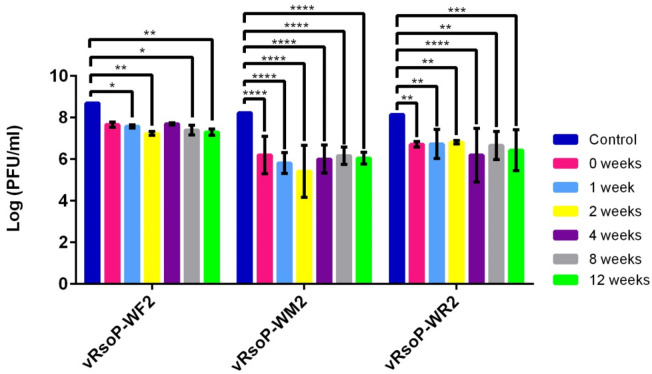
Stability of phages vRsoP-WF2, vRsoP-WM2 and vRsoP-WR2 lyophilized with trehalose at 0.5 M and stored at 4 °C over time. Titration was carried out by the double layer agar method on CPG plates. Data are the means for assays performed at least in triplicate, and error bars indicate variation as the standard deviation. Asterisks above bars denote statistically significant differences between the two signaled columns: * *p* < 0.05, ** *p* < 0.01, *** *p* < 0.001, **** *p* < 0.0001.

The viral suspensions were titrated before their lyophilization, as controls, and then also the obtained lyophiles at time zero and periodically for 12 weeks to determine the viability of the phages. As it can be seen in Figure 4, the three phages maintained their viability after lyophilization with trehalose but, this was lower than that of the controls. In the case of phage vRsoP-WF2, the titre initially decreased one logarithmic order, from 10^8^ to 10^7^ PFU/mL, then maintained over time. In phage vRsoP-WM2, the decrease was more pronounced, going from 10^8^ to 10^6^ and 10^5^ PFU/mL, and staying around the latter value. In the case of vRsoP-WR2, there was a reduction of two logarithmic units, from 10^8^ to 10^6^ PFU/mL, remaining at this level throughout the experimental period. Thus, the survival of phage vRsoP-WM2 was lower than that of the others, under the conditions tested. In most cases differences were statistically significant (*p* ≤ 0.05) between initial viability values and values obtained at each time, for each of the three phages (Figure 4). Among the three phages, differences were statistically significant (*p* ≤ 0.05) when comparing vRsoP-WM2 and vRsoP-WR2 with vRsoP-WF2 (Figure 4).

#### 3.2.2. Phage Stability after Lyophilization

To compare the stability of the lyophiles of the different phages with that of other conservation methods, at the same time that the lyophiles were prepared, phage suspensions from the same stocks were prepared in LB medium and SM buffer, and kept at 4 °C.

**Figure 5:**
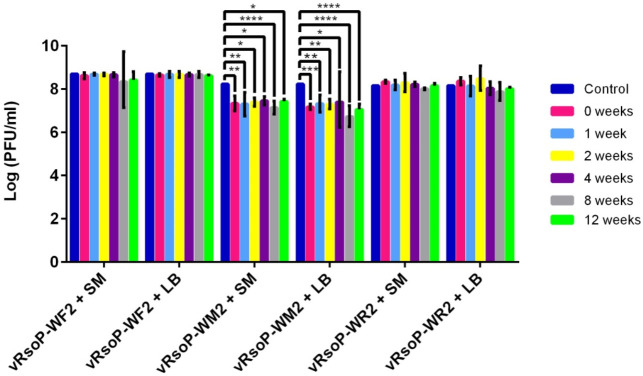
Stability of phage vRsoP-WF2, vRsoP-WM2 and vRsoP-WR2 suspensions preserved in LB medium and in SM buffer at 4 °C over time. Titration was carried out by the double layer agar method on CPG plates. Data are the means for assays performed at least in triplicate, and error bars indicate variation as the standard deviation. Asterisks above bars denote statistically significant differences between the two signaled columns: * *p* < 0.05, ** *p* < 0.01, *** *p* < 0.001, **** *p* < 0.0001.

Thus, each time the lyophilized phages were titrated, these phage suspensions at 4 °C were titrated in parallel. The results showed a greater stability and viability of the viral particles of these suspensions at 4 °C throughout the same experimental period (Figure 5). The viability of phages vRsoP-WF2 and vRsoP-WR2 was not reduced, contrarily to their viability as lyophiles for the same period (Figure 4); in fact, their titers maintained values around 10^8^ PFU/mL throughout the 12 weeks. However, the viability of the phage vRsoP-WM2 decreased one logarithmic order, from 10^8^ to 10^7^ PFU/mL, although this reduction was lower than that observed when lyophilized (Figure 4). Differences were statistically significant (*p* ≤ 0.05) between initial viability values and values obtained at each time only for the phage vRsoP-WM2 in both LB medium and SM buffer (Figure 5). For the other two phages, vRsoP-WF2 and vRsoP-WR2, these conservation methods did not reduce significantly their viability, at least during 12 weeks. Among the three phages, differences were statistically significant (*p* ≤ 0.05) mostly when comparing vRsoP-WM2 with vRsoP-WF2 and vRsoP-WR2 (Figure 5).

### 3.3. Effect of Lyophilization on the Lytic Activity of the Phages vRsoP-WF2, vRsoP-WM2 and vRsoP-WR2 against Different Bacterial Strains

To determine the maintenance of the lytic activity of the phages after their lyophilization at different times, *R. solanacearum* strains of various sources and geographical origins were used (Table 1) in qualitative spot assays with the nonlyophilized and the lyophilized phages.

The strain CFBP 4944 was used as a control, since it has been shown that it is lysed by the three phages, both the nonlyophilized and the lyophilized (Figure 4 and Figure 5). All the tested strains were lysed by the three phages before and after having been subjected to the lyophilization process. No differences were observed among the three phages prior to lyophilization, their titers were similar and the obtained lytic zones were of similar size with all tested strains. However, the phage vRsoP-WM2 was comparatively the most affected by the lyophilization, since it showed lower titers after this process, around one log unit, and thus smaller lytic zones against most of the tested strains, while lyophilized vRsoP-WF2 and vRsoP-WR2 maintained their activity against the different strains of *R. solanacearum* 1 and 4 weeks after lyophilization.

### 3.4. Bacterial Wilt Biocontrol Assays in Susceptible Plants Inoculated with R. solanacearum and Mixtures of vRsoP-WF2, vRsoP-WM2 and vRsoP-WR2

To determine the potential of the lyophilized phages to control the bacterial wilt disease, biocontrol assays were carried out with tomato plants inoculated simultaneously with the pathogen and the three phages lyophilized for 4 weeks comparatively to a mixture of the nonlyophilized. Strain CFBP 4944 was used in the assays, and the respective controls were included. Results were obtained as the percentage of plants with wilting symptoms with respect to the total number of inoculated plants.

**Figure 6:**
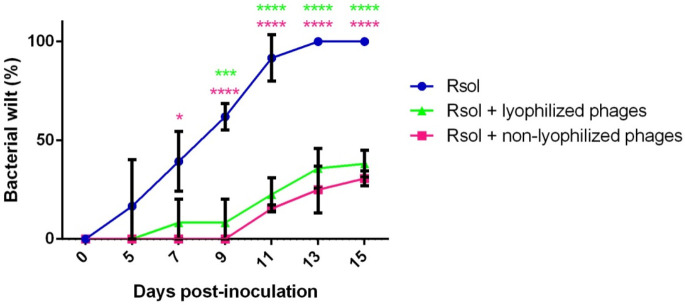
Bacterial wilt biocontrol assays *in planta* inoculated with *Ralstonia solanacearum* strain CFBP 4944 and a mixture of the phages vRsoP-WF2, vRsoP-WM2 and vRsoP-WR2 either nonlyophilized or lyophilized after 4 weeks. Mixtures were co-inoculated at final concentrations bacterium:phages of 10^5^ CFU/mL:10^8^ PFU/mL. Positive controls (Rsol) were at 10^5^ CFU/mL. Negative controls (mixtures of phages) were at 10^8^ PFU/mL. Additional negative controls were performed with PBS. Plants inoculated with negative controls, either mixtures of phages or PBS, did not develop wilting symptoms (not shown). Data are the means for assays performed in triplicate, and error bars indicate variation as the standard deviation. Asterisks denote statistically significant differences between the treatments: * *p* < 0.05, *** *p* < 0.001, **** *p* < 0.0001.

Plants treated with the bacterium and the phages showed a reduction in wilting symptoms with respect to plants inoculated only with the bacterium (Figure 6). The treated plants did not reach 100% wilting in any of the assays, contrarily to the positive controls, which completely wilted within the second week after inoculation. Wilting values for treated plants were around 33.3%, either those inoculated with the pathogen and a mixture of phages lyophilized for 4 weeks or a mixture of the nonlyophilized. Regarding the rate of appearance of bacterial wilt symptoms, an initial variation could be observed between the phages lyophilized for 4 weeks and the nonlyophilized (Figure 6), although at the end of the assay results were comparable, and it could be argued that the phages can prevent and control bacterial wilt almost equally. Negative control plants, inoculated with PBS or the nonlyophilized phages or the phages lyophilized for 4 weeks, remained asymptomatic with 0% wilting throughout the experimental period. Differences were statistically significant (*p* ≤ 0.05) between the inoculations with the pathogen and the pathogen with a mixture with either lyophilized or nonlyophilized phages. No significant differences were observed between the treatments with lyophilized and nonlyophilized phages (Figure 6).

Tomato plants with wilting symptoms were processed for *R. solanacearum* reisolation, and the pathogen was recovered on SMSA. The selected colonies with typical reddish coloration and morphology were PCR identified as *R. solanacearum*.

Phages were seldom detected within the first week but, after 15 days of inoculation, their detection was only possible in the control plants inoculated with nonlyophilized phages, where plaques could be observed on the plates. No plaque was detected from plants inoculated with the mixture of lyophilized phages, either with the mixture of bacteria and nonlyophilized phages, or the mixture of bacteria and phages lyophilized after 4 weeks, in the selection of plants analyzed.

## 4. Discussion

The growing interest in phages as biocontrol agents for bacterial diseases of plants makes it necessary to develop methods of phage preservation that are reliable in the medium and long term to allow their commercialization. One of the methods considered the most effective in terms of preserving the characteristics of the microorganisms and their ease of storage and transport is lyophilization, since it could allow both the maintenance of the viral particles and their readily distribution [22,26].

Since a biocontrol method of bacterial wilt based on the use of the phages vRsoP-WF2, vRsoP-WM2 and vRsoP-WR2 due to their lytic activity against *R. solanacearum* was developed and patented [28,29,30,31], the viability and stability of the three phages after subjecting them to an aggressive freeze-drying preservation process as the lyophilization became a necessary objective in view of their commercialization.

Lyophilization implies a strong stress for the viral particles, which can lead to a great loss of viability unless suitable cryoprotective or lyoprotective agents are used. Based on the literature [22,25,36,42], an initial assay was performed with the phage vRsoP-WF2 testing three cryoprotectants. One of them was 50% glycerol, which did not lyophilize coinciding with previous studies and the viability of vRsoP-WF2 dropped below the detection levels. This fact demonstrated that without the presence of an adequate cryoprotective agent, lyophilization is a very aggressive process able of destroying microorganisms. This has been related to the formation of ice crystals during freezing, in addition to the increase in osmolarity resulting from an increase in internal solute concentration, and to the denaturation of molecules due to dehydration [22,43]. On the contrary, when saccharose and trehalose, disaccharides described as good cryoprotectants in other phages [22,23,24,25,37,42,44,45] were used, the results revealed that both allowed to maintain high concentrations of viable viral particles at the two concentrations tested (0.1 M and 0.5 M), being higher at the highest concentration and in the presence of trehalose. In this case, the cryoprotection mechanisms provided by these two disaccharides have been described as involving vitrification, i.e., the conversion of a material to solid devoid of crystalline structures and, on the other hand, the formation of a matrix that prevents the aggregation of microorganisms and mechanical stress by ice crystals [22]. In addition, it has a low hygroscopicity due to the lack of internal hydrogen bonds, which allows for greater plasticity in the formation of these bonds on proteins [22,46] and, in this way, it can prevent the inactivation of phages during the lyophilization process [47].

Based on these results, the assay was repeated only with 0.5 M saccharose and 0.5 M trehalose, confirming the results of the first assay and thus trehalose as the best cryoprotective agent for vRsoP-WF2, coinciding with previous studies on other phages [22].

On the other hand, the stability of vRsoP-WF2 was monitored over time in order to evaluate medium to long term preservation. The results showed higher stability in the presence of trehalose 0.5 M than in saccharose during 60 days after lyophilization. These differences were not very pronounced but biologically relevant, and they could be marked at longer times, so it would be necessary to continue the study in the longer term.

When these results were compared with those obtained with controls maintained in LB medium and in SM buffer at 4 °C, it was observed that the stability was greater for these controls within the experimental period, and pronounced losses of viability as those obtained with the lyophilization process did not occur. However, lyophilization has several advantages, such as the smaller volume of sample to be preserved as opposed to the larger volumes of microbial suspensions in liquid media; greater ease of transport and shipping; lower maintenance costs because it does not require subzero temperatures; and a lower risk of contamination, since lyophilized viral particles are dehydrated and in vacuum conditions, unlike stocks of viral suspensions in liquid media.

Based on the results abovementioned, the effect of lyophilization on the viability and stability of vRsoP-WF2, vRsoP-WM2 and vRsoP-WR2 was addressed using 0.5 M trehalose. The viability of the three phages after lyophilization tended to slight decreases with respect to the nonlyophilized controls. Thus, phages vRsoP-WF2 and vRsoP-WR2 presented reduced viability values, around 1 and 1.5 logarithmic units, and the phage vRsoP-WM2 reductions of around 2 and 3 logarithmic units, with respect to the controls and under the conditions tested. Among the three phages, vRsoP-WM2 seemed to be more affected by the lyophilization than vRsoP-WF2 and vRsoP-WR2, possibly because it is more sensitive to the desiccation and/or freezing treatments suffered during the process, or maybe trehalose at the concentration tested is not the best cryoprotectant for this phage, hindering adequate preservation and survival.

Likewise, also after lyophilization, the titers of the three phages decreased in comparison with their respective controls, even if a cryoprotectant was added [36,48]. In contrast, the titers of the phages that were directly conserved in LB medium and in SM buffer at 4 °C did not decrease, except for vRsoP-WM2. Therefore, this phage exhibits greater sensitivity to conservation at low temperature than the other two phages, similarly to what happened in relation to conservation by lyophilization. In this regard, the genomic characterization of these three phages has been recently reported [49] showing that their genomes are very closely related among them (>99%), although with minor insertions and deletions, except for vRsoP-WR2, which also possesses a large insertion absent in the other two phages. They all share genomic organization with only 22 or 23 predicted proteins with functional homologs in databases. The two most similar phages are vRsoP-WF2 and vRsoP-WM2. Therefore, the differences observed in the stability of the three phages after lyophilization with 0.5 M trehalose do not seem to be related to genetic differences. Overall, even with the exception of vRsoP-WM2, the phages preserved at 4 °C have remained more stable than lyophilized, at least for a period of three months. In fact, phages can be kept stable for long periods under low temperatures [21,44,46,50]. However, lyophilization has other valuable advantages already mentioned.

The biocontrol potential of the phages after lyophilization in susceptible host plants was evaluated. To verify the efficacy of a cocktail of the three *R. solanacearum* phages after lyophilization, biocontrol assays were carried out in tomato plants co-inoculated with the pathogen and a cocktail of phages either lyophilized or not. In the assays, biocontrol percentages were higher than 65% in the plants treated with the nonlyophilized phage cocktail, whereas the biocontrol percentages with the cocktail of lyophilized phages at 4 weeks were higher than 50%. In contrast, the plants inoculated only with *R. solanacearum* presented wilting symptoms earlier than those treated with phages, with 100% wilting in less than 2 weeks. Plants treated only with phages did not develop any disease symptoms, indicating their safety and suitability as biocontrol agents. Up until now, there are no other references on the effect of a conservation method as the lyophilization on the biocontrol efficacy *in planta* of *R. solanacearum* phages.

In summary, the work carried out has made possible to increase the knowledge on the viability and stability of lyophilized and nonlyophilized phages vRsoP-WF2, vRsoP-WM2 and vRsoP-WR2, and on their interaction with *R. solanacearum.* It has been found that they remain fairly stable when lyophilized with trehalose 0.5 M, at least during a period of 90 days. Further, the three phages retain their lytic activity against *R. solanacearum* and biocontrol efficacy in susceptible tomato plants, providing determination of their preventive potential or therapeutic action on the bacterial wilt disease.

## Figures and Tables

**Table 1:** Strains of *Ralstonia solanacearum* used in the present study.

Strain Code	Phylotype	Race	Biovar	Host	Country
CFBP ^1^ 4944	2	3	2	*Solanum tuberosum*	Spain
IVIA ^2^ 1496-19, 1632-2, 1670, 1674	2	3	2	*S. tuberosum*	Spain
IVIA 2297 4T2a	2	3	2	Soil	Spain
EURS ^3^ 48	2	3	2	*S. lycopersicum*	United Kingdom
EURS 67	2	3	2	*S. tuberosum*	Belgium
EURS 71	2	3	2	*S. tuberosum*	France
SMT ^4^ 46	ND ^5^	1	3	*S. lycopersicum*	Peru
203 Mexico	2	3	2	*S. lycopersicum*	Mexico

^1^ CFBP: French Collection of Phytopathogenic Bacteria, ^2^ IVIA: Valencian Institute for Agricultural Research (Spain), ^3^ EURS: European Project CE-FAIR-PL97-3632, ^4^ SMT: European Project CE-SMT4-CT97-2179, ^5^ ND: not determined.

## Data Availability

Data is contained within the article.
